# The Innate Cellular Immune Response in Xenotransplantation

**DOI:** 10.3389/fimmu.2022.858604

**Published:** 2022-03-28

**Authors:** Akira Maeda, Shuhei Kogata, Chiyoshi Toyama, Pei-Chi Lo, Chizu Okamatsu, Riho Yamamoto, Kazunori Masahata, Masafumi Kamiyama, Hiroshi Eguchi, Masahito Watanabe, Hiroshi Nagashima, Hiroomi Okuyama, Shuji Miyagawa

**Affiliations:** ^1^Department of Promotion for Blood and Marrow Transplantation, Aichi Medical University School of Medicine, Nagakute, Japan; ^2^Department of Pediatric Surgery, Osaka University Graduate School of Medicine, Suita, Japan; ^3^International Institute for Bio-Resource Research, Meiji University, Kawasaki, Japan

**Keywords:** xenotransplantation, macrophage-mediated xenogeneic rejection, innate cellular response, NETosis, ITIMs

## Abstract

Xenotransplantation is very attractive strategy for addressing the shortage of donors. While hyper acute rejection (HAR) caused by natural antibodies and complement has been well defined, this is not the case for innate cellular xenogeneic rejection. An increasing body of evidence suggests that innate cellular immune responses contribute to xenogeneic rejection. Various molecular incompatibilities between receptors and their ligands across different species typically have an impact on graft outcome. NK cells are activated by direct interaction as well as by antigen dependent cellular cytotoxicity (ADCC) mechanisms. Macrophages are activated through various mechanisms in xenogeneic conditions. Macrophages recognize CD47 as a “marker of self” through binding to SIRPα. A number of studies have shown that incompatibility of porcine CD47 against human SIRPα contributes to the rejection of xenogeneic target cells by macrophages. Neutrophils are an early responder cell that infiltrates xenogeneic grafts. It has also been reported that neutrophil extracellular traps (NETs) activate macrophages as damage-associated pattern molecules (DAMPs). In this review, we summarize recent insights into innate cellular xenogeneic rejection.

## The Pivotal Role of Innate Immunity in Xenogeneic Rejection

While the main cells that infiltrate allograft rejections are cytotoxic T lymphocytes (CTL); xenografts induce the infiltration of NK cells, macrophages and neutrophils (1).

Neutrophils are the most abundant circulating leukocytes and a cell population that responds early to infiltrate cellular and solid organ xenografts following transplantation (2, 3). These cells are subsequently replaced by macrophages and T cells (2, 3).

**Figure 1:**
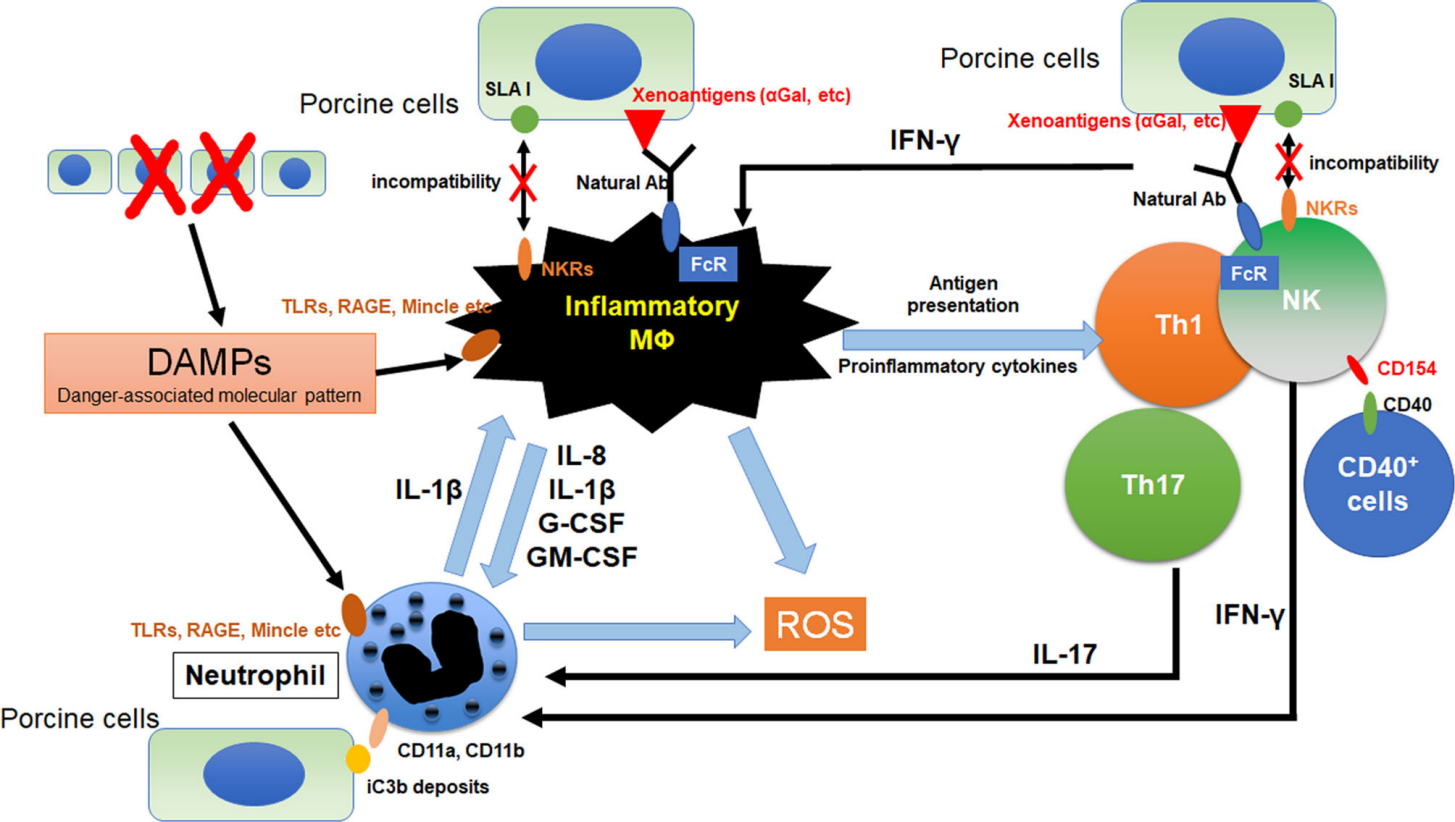
Crosstalk between macrophages and other innate immune cells in xenograft. The immunocomplex of porcine cells and natural antibodies against porcine antigens binds to Fc receptors (FcRs) on macrophages and NK cell. Activated NK cell and macrophage induce antibody-dependent cellular cytotoxicity (ADCC) against porcine cells. Furthermore, incompatibility between SLA1 and NK receptors (NKRs) causes macrophage and NK cell activation because of lack of inhibitory signals. Macrophages were activated by the binding of DAMPs from dead cells to toll-like receptors (TLRs), receptor for advanced glycation end-products (RAGE) and macrophage-inducible C-type lectin (Mincle). Activation signals were induced and various pro-inflammatory cytokines are released from macrophages. These cytokines activate neutrophils and promote NETosis. Especially, IL-8 contributes to the neutrophil recruitment and IL-1β enhances NETosis formation in neutrophils. In addition, NETs from neutrophils also activate macrophages as DAMPs. Macrophages can activate NK cells and IFN-γ from activated NK cells was known to activates both macrophage and neutrophil. CD11a and CD11b on neutrophils bind to iC3b deposits on porcine cells, suggesting that neutrophils can recognize porcine target cells *via* binding of CD11a,b and iC3b. CD154 on NK cells enhances NK cytotoxicity to CD40 positive target cells.

Neutrophils induce tissue damage under xenogeneic conditions in both antibody-dependent and antibody-independent manners (4–6). In the response to inflammatory stimuli, neutrophils induce a unique type of cell death process that is referred to as “NETosis”. They release network structures that are referred to as neutrophil extracellular traps (NETs), and contains serine proteases and antibacterial peptides. NETs themselves induce tissue damage through the generation of reactive oxygen species (ROS) (7–10) and the release of digestive enzymes (11, 12). Moreover, NETs are recognized by macrophages as damage-associated molecular patterns (DAMPs) which triggers inflammatory signals and IL-1beta production in macrophages (12). It has also been reported that the IL-1beta produced by macrophages enhances NET formation in neutrophils (13). Because macrophages are also activated under xenogeneic conditions and directly reject a xenograft (14, 15), inhibiting this negative chain reaction by inhibiting the action of neutrophils leads to the suppression of innate cellular rejection in a xenograft (**Figure 1**).

Macrophages are activated *via* various mechanisms under xenogeneic conditions through various mechanisms. As an antibody-dependent mechanism, immunocomplexes of porcine cells with xenogeneic antigen-specific antibodies such as anti-α1,3Gal natural antibodies bind to FcγR and induce the production of an activation signal (16, 17). As an antibody-independent mechanism, the binding of galectin-3 to α1,3Gal on porcine cells has been reported to function as an activation signal in macrophages (18–20). Macrophages are also activated by interactions with neutrophils, NK cells and Th1 cells (21, 22). DAMPs arising from damaged porcine cells also contribute to macrophage-mediated rejection (23). Activated macrophages induce tissue damage in grafts *via* the secretion of various proinflammatory cytokines, reactive oxygen and nitrogen species, and complement factors (24).

NK cells rapidly infiltrate porcine xenografts that have been perfused ex vivo with human blood and reject xenogeneic cells by direct interaction or by antibody dependent cellular cytotoxicity (ADCC) mechanisms. NK cells which are activated in an antibody-independent manner, induce cytotoxicity mainly by the release of perforin and granzymes (25–27). The direct cytotoxicity of NK cells is regulated by the balance between the activation and the inhibition of signal pathways that are mediated by various NK receptors (28). The activating NK receptors NKG2D (29) and pULBP-1 (30) bind to the porcine ligand NKp44 and an unidentified molecule, respectively, resulting in the release of lytic granules. However, the inhibitory receptors on human NK cells, KIR, ILT2, and CD94/NKG2A, do not efficiently identify swine leukocyte antigen I (SLAI, swine MHC class I), leading to invalidating inhibitory signals for NK cell activation (31, 32). The deposits of natural and elicited antibodies on the graft endothelial cells are recognized by the Fc-receptors (FcRs) on NK cells. Interaction between FcRs and immunocomplex of porcine cell and antibody causes the release of perforin and granzymes from NK cells and, in turn, triggers target cell apoptosis (33). In addition to xenoantibodies that are bound on the endothelial cells, the anti-swine leukocyte antigen class I (anti-SLA classI) antibodies also induce antibody-dependent cellular cytotoxicity (ADCC) (28, 34).

## Inhibition of NK Cell-Mediated Xenograft Rejection

NK cells are activated in antibody-dependent and -independent manners under xenogeneic conditions. The knock out of alpha1,3-galactosyltoransferase (αGalT) is known to suppress NK-mediated ADCC but not antibody independent cytotoxicity (35, 36). NK cells are known to be regulated by the balance between activation signals and inhibitory signals and receive inhibitory signals from MHC class I molecules. In the case of xenografts between humans and pigs, the inhibitory receptors on NK cells fail to deliver inhibitory signals due to the lack of human leukocyte antigens (HLA) on target cells. To protect porcine target cells from being killed by human NK cells, classical and non-classical human MHC class I molecules were forcibly expressed in porcine cells and, in a number of studies, have been reported to suppress NK xenocytotoxicity (37–40). HLA-G_1_ is recognized by CD158d and immunoglobulin-like transcripts (ILT) -2,4. It is known that HLA-E functions as a ligand for the CD94/NKG2A inhibitory receptor and the CD94/NKG2C activation receptor. These receptors have inhibitory immunoreceptor tyrosine-based inhibitory motifs (ITIMs) in their cytoplasmic tail and induce inhibitory signal in NK cells. Therefore, the modification of HLA-G_1_ and HLA-E cDNA for transfection for it to only bind to inhibitory receptors would be expected to improve the efficacy of non-classical HLA strategies.

An anti-CD154 monoclonal antibody, a co-stimulation blockade agent, has recently been reported to be effective in xenogeneic rejections (41, 42). IL-2-activated human NK cells express CD154 (the CD40 ligand, CD40L) and the crosslinking of CD154 on NK cells enhances the NK cytotoxicity to CD40 positive target cells (43). NK cells also contribute to antibody-mediated rejection by interaction with marginal zone B cells in the spleen *via* the CD40-CD154 pathway and the production of T cell-mediated αGal-independent antibodies (44). These findings indicate that anti-CD154 mAb may also suppress NK-mediated xenogeneic rejection. We recently demonstrated that prenylated quinolinecarboxylic acid (PQA)-18, a p21-activated kinase 2 (PAK2) inhibitor, suppresses the expression of CD40 on macrophages (45). A combination therapy of anti-CD154mAb and PQA-18 would therefore be expected to induce a greater suppression in xenogeneic rejection.

## Inhibition of Macrophage-Mediated Xenograft Rejection

CD47-SIRPα has been reported to play a critical role in signaling by virtue of its ability to prevent phagocytosis (46–48). CD47 binds to SIRPα and prevents the clearance of cells by the immune system. SIRP-α is expressed by macrophages and neutrophils and recognizes CD47 as a marker of self. The cytoplasmic domain of SIRP-α contains ITIMs that function to regulate their immunological function by recruiting the tyrosine phosphatase Src homology 2 domain-containing protein tyrosine phosphatase-1 (SHP-1). CD47-SIRPα signaling acts as a “do not eat me” signal and reduces the macrophage-mediated phagocytosis of opsonized cells. In addition, a large number of studies have shown that pig to human molecular incompatibility of SIRPα-CD47 interactions contribute to the rejection of xenogeneic cells by macrophages (49–53). The ectopic expression of human CD47 on swine endothelial cells has been reported to lead to significant reduction of the human macrophage-mediated phagocytosis of xenogeneic cells. However, a recent study by Chen et al. reported that thrombospondin (TSP)-1-CD47 signaling may stimulate vascularized allograft rejection (54). TSP-1 also binds to CD47 and inhibits the binding of SIRP-α to CD47 in macrophages. TSP-1 blocks hCD47- SIRP-α signaling and increases phagocytosis in macrophages. Moreover, TSP-1 contributes to platelet aggregation (55) and up-regulates the expression of TSP-1 in ischemic tissues and damaged allografts, resulting in vasculopathy and rejection (56–58). The strong and week points of CD47 in xenografts will likely be revealed in future studies.

Human inflammatory monocyte-derived macrophages that are generated from peripheral blood monocytes express CD94/NKG2A and ILT-2,4, which are inhibitory NK receptors for HLA-E and G_1_ (59, 60). The expression of both transgenic HLA-E and HLA-G_1_ on porcine cells significantly suppresses macrophage-mediated xenogeneic cytotoxicity. Furthermore, both HLA-E and G_1_ were reported to suppress the production of proinflammatory cytokines by macrophages. Because non-classical MHC class I molecules suppress both macrophage and NK rejection, generating HLA-E and G_1_ transgenic pigs would be an excellent strategy for inhibiting innate cellular rejection.

It was recently reported that the overexpression of human CD200 on porcine cells suppresses xenogeneic macrophage-mediated cytotoxicity and phagocytosis (61, 62). CD200 and its receptor, CD200R, are both members of the immunoglobulin superfamily. CD200 induces immunosuppression *via* binding to CD200R, which contains inhibitory NPXY signaling motifs in the cytoplasmic region. The suppression of allogeneic graft rejection by CD200-CD200R signaling has been reported in animal models (63, 64). CD200 on porcine cells not only suppresses macrophage-mediated phagocytosis and cytotoxicity, but also suppresses the release of proinflammatory cytokines from macrophages under xenogeneic conditions. It has been reported that the recombinant fusion protein CD200-Ig (OX2) functions to prolong allograft survival (65). The function of OX2 is expected to be confirmed in a xenogeneic model.

The surfactant proteins (SP)-A and SP-D are members of a family of molecules that induce innate immune responce as pathogen-associated molecular patterns (PAMPs) (66, 67). On the contrary, it has also been reported that SP-A and -D have anti-inflammatory properties (68, 69). While the ligation of the N-terminal collagen domains of SP-A and -D with the calreticulin/CD91 receptor complex induce inflammation, the binding of the C-terminal heads to SIRPα induces an inhibitory signal in innate immune responses (70, 71). We recently demonstrated that the forcible expression of carbohydrate recognition (CRD) of SP-D on porcine endothelial cells significantly suppresses macrophage-mediated cytotoxicity and the production of proinflammatory cytokine from macrophages (72). Because SP-A and D also bind to SIRPα on neutrophils, SP-A and D may also suppress neutrophil-mediated xenogeneic rejection.

Cellular xenografts are rejected predominantly by cellular immune responses. This response is initiated by CD4+ T cells (73–76) and macrophages have been reported to be the most important effector cells that are involved in this response (77). The JAKs inhibitor, Tofacitinib, has been reported to inhibit the production of macrophage-mediated inflammatory cytokines (78, 79). In addition, Kim et al. reported that Tofacitinib suppress allogenic rejection in islet transplantation (80). We recently reported that both PQA-18, a PAK2 inhibitor, and Tofacitinib suppress both the differentiation of macrophages and xenogeneic macrophage-mediated cytotoxicity (45). Furthermore, both PQA-18 and Tofacitinib suppress the induction of xenogeneic cytotoxic T lymphocytes (CTLs) to a considerable extent in mixed lymphocyte reactions (MLR). These findings suggest that PQA-18 and Tofacitinib suppress not only innate immunity but also acquired immunity under xenogeneic conditions and may be effective in both cellular and organ xenograft rejection.

## Inhibition of Neutrophil-Mediated Xenogeneic Rejection

A number of studies dealing with human neutrophil migration and adherence to pig endothelial cells have been reported (81–83). al-Mohanna et al. reported that the binding of neutrophils to xenogeneic cells is dependent on the interaction between CD11a and Mac-1 (CD11b/CD18, an iC3b receptor) and their ligands for CD11a and CD11b/CD18 suppress neutrophil adhesion *in vitro* (84). MacNally et al. It is known that activated neutrophils are attached to foreign surfaces through the interaction of CD11b/CD18 with surface-bound iC3b (85), suggesting that neutrophils are able to recognize xenogeneic cells *via* the binding iC3b to CD11b/CD18 on neutrophils. A recent study by Bastian et al. supported this finding. They found that, when decellularized porcine heart valveswere incubated in plasma, iC3b was deposited and a significant adhesion of neutrophils was observed (86). CD82, one of the tetraspanins, was also reported to contribute to the adhesion of human neutrophils to porcine cells (87). Saleh et al. reported that CD82 contributes to the Galα1,3-Gal-independent adhesion of neutrophils to porcine endothelial cells. Indeed, the compstatin analogue Cp40, a potent complement C3 inhibitor, was found to suppress CD11b expression on neutrophils and neutrophil adhesion to xenogeneic target cells (88).

Wang et al. recently reported that the ectopic expression of human CD31 on porcine cells suppress xenogeneic neutrophil NETosis and cytotoxicity (89). CD31 (PECAM-1) is well known as an adhesion molecule and is ubiquitously expressed by various inflammatory cells (90). CD31 homophilic ligation induces signal transduction *via* its immunoreceptor tyrosine-based inhibition motif (ITIMs) to attenuate tyrosine kinase-mediated signaling pathways (91–93). Because CD31 has been reported to be incompatible between human and pig (94, 95), human CD31 may be a good candidate for suppressing neutrophil-mediated xenogeneic rejection.

## Conclusion

The infiltration of various inflammatory leukocytes into α1,3-galactosyltransferase knockout (αGalTKO) neonatal porcine islets (96), which suggests that suppressing antibody dependent rejection is not sufficient to prevent xenogeneic cellular innate immunity. Therefore, a strategy that involves the suppression of direct recognition by innate immune cells is essential for clinical applications of xenografts. A variety of strategies for suppressing NK and macrophage-mediated rejection have been proposed, but there appears to be quite a bit of room for improvement. Neutrophils contribute, not only to the induction of inflammation but also to the resolution of inflammation. The phagocytosis of apoptotic cells by macrophages plays a pivotal role in the resolution of an inflammatory response and apoptotic neutrophils themselves exert anti-inflammatory effects (97, 98). The Bcl-2 homologue, Mcl-1, which functions to prevent intrinsic apoptosis, is central to the ability of neutrophils to undergo rapid apoptosis (99). Hence the induction of a Bcl-2 proapoptotic homologue such as Bax in important in terms of inducing apoptosis of neutrophils. The death receptor ligand, TRAIL, has been reported to induce apoptosis in neutrophils (100). A leucine zipper-tagged form of TRAIL may therefore be a good strategy for induce the resolution of inflammation.

**Figure 2:**
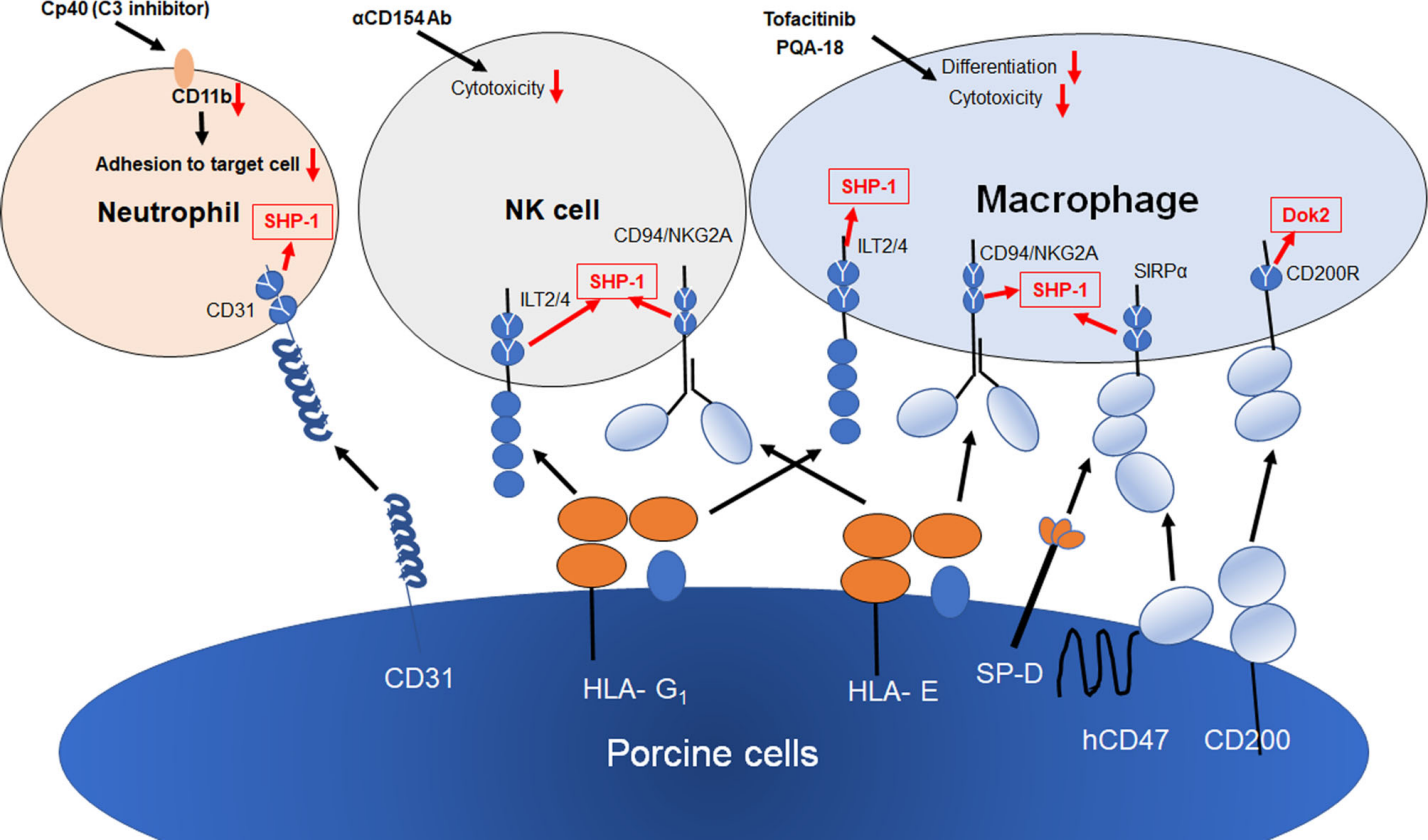
Strategies for innate cellular xenogeneic rejection. Both HLA-E and G1 on porcine endothelial cells suppress NK and macrophage-mediated xenogeneic rejection. The transgenic expression of human CD47, SP-D and CD200 on porcine cells results in the suppression of macrophage-mediated cytotoxicity and inflammation. Human CD31 inhibits NETosis in neutrophils *via* the homophilic binding to CD31 on neutrophils. Both Tofacitinib (JAK inhibitor) and PQA-18 (PAK2 inhibitor) have been reported to suppress macrophage-mediated cytotoxicity and differentiation of macrophages. Anti-CD154 Ab suppress NK cytotoxicity against CD40^+^ target cells. Cp40 (C3 inhibitor) suppress CD11b expression, resulting in suppression of neutrophil adhesion to porcine cell. Y shows an inhibitory intracellular signaling motif of inhibitory receptors.

In conclusion, significant advances have been made in the field of xenogeneic cellular immune responses in the past 20-30 years (101). Various transgenic and knockout pigs were generated to suppress the xenogeneic rejection. The strategies against innate cellular xenogeneic rejection were summarized in **Figure 2**. Further improvements will be needed if better effects are to be achieved in the future.

## Author Contributions

Collection of manuscript was done by AM while SK, CT, P-CL, CO, RY, KM, MK, HE, and MW collected relevant literature. HN, HO, and SM edited and approved of the manuscript. All authors contributed to the article and approved the submitted version.

## Funding

This work was supported by Grants-in Aid for Scientific Research, and Health and Labor Sciences Research Grants, Japan (grant number is 19K9092).

## Conflict of Interest

The authors declare that the research was conducted in the absence of any commercial or financial relationships that could be construed as a potential conflict of interest.

## Publisher’s Note

All claims expressed in this article are solely those of the authors and do not necessarily represent those of their affiliated organizations, or those of the publisher, the editors and the reviewers. Any product that may be evaluated in this article, or claim that may be made by its manufacturer, is not guaranteed or endorsed by the publisher.
